# Exploring Peptide–Solvent Interactions: A Computational Study

**DOI:** 10.3390/molecules23092355

**Published:** 2018-09-14

**Authors:** Nadia Elghobashi-Meinhardt

**Affiliations:** Theoretical Molecular Biophysics, Department of Physical and Theoretical Chemistry, Institute for Chemistry and Biochemistry, Freie Universität Berlin, Fabeckstr. 36a, 14169 Berlin, Germany; nadia.elghobashi-meinhardt@fu-berlin.de; Tel.: +49-30-838-52128

**Keywords:** peptides, aqueous solvent, XAO peptide, molecular dynamics (MD), quantum mechanics/molecular mechanics (QM/MM), polyproline II

## Abstract

The dilemma of reconciling the contradictory evidence regarding the conformation of long solvated peptide chains is the so-called “reconciliation problem”. Clues regarding the stability of certain conformations likely lie in the electronic structure at the peptide–solvent interface, but the peptide–solvent interaction is not fully understood. Here, we study the influence of aqueous solvent on peptide conformations by using classical molecular dynamics (MD) and quantum mechanical/molecular mechanical (QM/MM) energy calculations. The model systems include an 11-residue peptide, X${}_{2}$A${}_{7}$O${}_{2}$ (XAO), where X, A, and O denote diaminobutyric acid, alanine, and ornithine, respectively, and a 9-mer (Arg-Pro-Pro-Gly-Phe-Ser-Ala-Phe-Lys). Spectroscopic and MD data present conflicting evidence regarding the structure of XAO in water; some results indicate that XAO adopts a polyproline II (P${}_{\mathrm{II}}$) conformation, whereas other findings suggest that XAO explores a range of conformations. To investigate this contradiction, we present here the results of MD simulations of XAO and the 9-mer in aqueous solution, combined with QM/MM energy calculations.

## 1. Introduction

**Figure 1:**
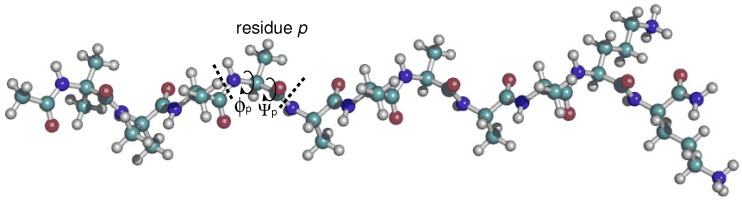
The 11-residue peptide X${}_{2}$A${}_{7}$O${}_{2}$ peptide (XAO), where X, A, and O denote diaminobutyric acid, alanine, and ornithine, respectively, contains seven consecutive alanine residues.

The small, water-soluble XAO peptide, X${}_{2}$A${}_{7}$O${}_{2}$ (XAO), where X, A, and O denote diaminobutyric acid, alanine, and ornithine, respectively, has been the subject of several experimental and theoretical studies that deliver contradictory results regarding the amount of local structure in the unfolded state of peptides. Particularly, quantifying the amount of locally ordered polyproline II (P${}_{\mathrm{II}}$) structure in an ensemble of states has proven to be a tricky issue. P${}_{\mathrm{II}}$ is a left-handed helix with 3.0 residues per turn, and backbone torsion angles of $\Phi \approx {-75}^{\circ }$ and $\Psi \approx {+145}^{\circ }$ [1] (see Figure 1). Proteins containing P${}_{\mathrm{II}}$ structure, typically arising from the presence of proline and glycine [2], have been associated with biological activities including signal transduction, transcription, and immune response [3].

Several studies have attempted to determine the amount of P${}_{\mathrm{II}}$ that exists in an XAO ensemble. Shi et al. carried out NMR and circular dichroism spectroscopy experiments and concluded that XAO in water adopts the P${}_{\mathrm{II}}$ conformation [4]. Kentsis et al. corroborated this finding with force field calculations [5]. On the other hand, more recent NMR, CD, calorimetry, and small angle X-ray scattering (SAXS) measurements of solvated XAO indicate that the P${}_{\mathrm{II}}$ conformation is only one of several structures present in the conformational ensemble that also includes α-helical and β-strand character [2,6,7,8]. These experimental data provoke the questions: what is the amount of P${}_{\mathrm{II}}$ structure in the conformational ensemble of XAO, and what are the interactions stabilizing the P${}_{\mathrm{II}}$ structure?

The dilemma of reconciling the contradictory evidence regarding the conformation of XAO and other solvated peptide chains is the so-called “reconciliation problem” [9]. Despite the efforts to solve this problem [9], controversies still exist [10]. Clues to unraveling the reconciliation problem are likely to lie in the peptide–solvent interactions. If one considers the P${}_{\mathrm{II}}$ structure, which is not a favored conformation in vacuum, but, according to some experimental findings, seems to be present in an ensemble of different conformations in solution, one can assume that the solvent plays a decisive role in stabilizing the peptide structure. In fact, the importance of solvent interactions in stabilizing specific protein conformations has long been recognized [11]. In a theoretical study of *N*-Methylacetamide, solvated in explicit water, the molecular properties, e.g., bond lengths, calculated with ab initio methods differed significantly from those obtained with an empirical model [12]. The complexity of the peptide–solvent interface was also probed in an experimental study in which solvated formamide was used to model a peptide residue in aqueous solution. In their study, Blanco et al. report clear evidence of σ-bond cooperativity; they observe the shortening and strengthening of hydrogen bonds between formamide and aqueous solvent molecules in large solvent-bridged structures, relative to 1:1 adducts [13]. The central question can thus be formulated: How does the presence of solvent dictate the conformation of an unfolded peptide?

To investigate this question, we performed 900 ns classical MD simulations of the XAO peptide in aqueous solution. We also carried out a shorter (200 ns) MD simulation of another small polypeptide chain, a 9-mer (Arg-Pro-Pro-Gly-Phe-Ser-Ala-Phe-Lys), a sequence that is often used as a fluorogenic peptide substrate and was also recently used as the peptide substrate in MD simulations of the eukarytoic proteasome core particle [14]. From the simulation data, we have analyzed torsional angle distributions, end-to-end lengths, and R${}_{\mathrm{gyr}}$. From snapshots of peptide conformations from the classical MD trajectory, we have then performed quantum mechanical (QM)/molecular mechanical (MM) energy calculations of the peptide with a small number of solvent molecules, in which the peptide atoms comprise the QM region. The results of our investigations provide some insight into the role of the aqueous environment in driving conformational changes in small peptides.

## 2. Methods

### 2.1. Preparation of XAO and 9-Mer Polypeptides

CHARMM (version 39a1) with the charmm36 all-atom topology and parameter files [15] was used to prepare the XAO peptide and the 9-mer. Interfaced with the semi-empirical quantum mechanical module SCC-DFTB (discussed in more detail below), CHARMM affords the ease of performing MM, QM, and hybrid QM/MM computations [16]. The charmm36 all-atom force field parameters, refined against a range of theoretical and experimental data on small peptides, shows improvement in the structural and dynamical behavior of backbone and side-chain moieties [17]. Importantly, charmm36 parameters are based on grid-based energy correction map (named CMAP) terms for protein backbone Φ,Ψ dihedral angles and side-chain torsion potentials [18]. This improvement corrects for the propensity of previous versions (e.g., charmm22) to overstabilize helices. Refinement of charmm36 parameters was carried out with both condensed phase and gas-phase QM calculations [19]. The force field has been validated against other state-of-the-art protein force fields, including AMBER ff03* [19], and it demonstrates improvements in reproducing a number of experimental observables at room temperature, including NMR and SAXS data [17]. Nonetheless, as with most force fields, the CHARMM force field was developed to target folded proteins, so its accuracy in describing unfolded proteins should be scrutinized [20]. Efforts to improve the charmm36 force field to describe intrinsically unfolded proteins are underway [20].

For the non-standard residues X (side chain –CH${}_{2}$–CH${}_{2}$–NH${}_{3}^{+}$) and O (side chain –[CH${}_{2}$]${}_{3}$–NH${}_{3}^{+}$), we followed the protocol of Zagrovic et al., i.e., modifying the charges of Lys by hand to distribute the extra charge throughout the C${}_{\beta }$H2 group in X; charges that were the same in Lys and Orn were kept unchanged [7]. The side chains were protonated according to Zagrovic et al. [7], i.e., assuming low pH, and the N- and C-termini were capped with acetyl and amide groups, respectively.

For the simulations performed in aqueous solution, neighboring dihedral angles were randomly chosen by CHARMM [16]. The XAO peptide was solvated with TIP3 water [21] and a simulation box was prepared with size 35×35×60 Å${}^{3}$ that included the XAO peptide and 2509 TIP3 water molecules. The system was prepared with four chloride anions to neutralize the net positive charge. An additional polypeptide chain was constructed, the 9-mer (Arg-Pro-Pro-Gly-Phe-Ser-Ala-Phe-Lys); the standard capping groups NH${}_{3}^{+}$ and COO${}^{-}$ were chosen for the terminal groups. The 9-mer was placed in a square water box (volume of 60 Å${}^{3}$; 7578 TIP3P water molecules [21]). The geometries of the solvated XAO and 9-mer peptides were optimized with 100 steps of steepest descent energy minimization, followed by 100,000 steps of adopted basis Newton–Raphson (ABNR) minimization with a gradient limit value of 0.01 kcal/mol/Å.

### 2.2. MD Simulations

MD simulations in water were carried out with NAMD [22] using the charmm36 all-atom topology and parameter files [15]. The MD simulations used a time step of 2 fs and the SHAKE algorithm [23] to constrain all bonds involving hydrogen atoms. Periodic boundary conditions were applied. A nonbonded cutoff of 10.0 Å was used and the nonbonded pair list was updated every 10 time steps. The temperature (300 K) was controlled using Langevin dynamics, with a collision frequency of 1.0 ps${}^{-1}$ and isotropic position scaling to maintain pressure (1 atm) [24]. The system was heated slowly in 10 K increments from 0 to 300 K over 60 ps. Data of production dynamics for XA0 and the 9-mer were collected for 900 ns and 200 ns, respectively. MD simulations of the XAO peptide in vacuo were carried out for 200 ns, following a similar heating and production protocol as for the XAO peptide in aqueous solvent. Convergence of production data for simulations in aqueous solution was checked by comparing ensemble averages of the first half of the trajectory to averages from the second half of the trajectory. These data are included in the Supplementary information.

For Φ,Ψ angle pairs, defined by C${}_{i-1}$–N${}_{i}$–C${}_{\alpha i}$–C${}_{i}$ and N${}_{i}$–C${}_{\alpha i}$–C${}_{i}$–N${}_{i+1}$ atoms, respectively, of adjacent peptide residues were evaluated over the total simulation time. For XAO in water, with 11 dihedral angle pairs evaluated over 900 ns, a total of 11×18148 = 199628 data points were generated; for XAO in vacuo, 100,000 data points were collected over 200 ns. For the 9-mer, with eight dihedral angle pairs evaluated over 200 ns, a total of 16000 data points were generated. The geometries were then assigned to six structural basins ($\alpha_{R}$, $\alpha_{L}$, 3${}_{10}$, C7${}_{\mathrm{eq}}$, β, P${}_{\mathrm{II}}$) in the Ramachandran plot [25], according to torsional angles listed in Table 1.

### 2.3. QM/MM Energy Function

The QM/MM computations were performed using a Quantum Mechanical/Molecular Mechanical (QM/MM) Hamiltonian [26,27,28]. In the QM/MM approach, the region of chemical importance is treated with QM, and the remainder of the system is described with MM. The total energy is given by

(1) $$E=E_{QM}+E_{MM}+E_{QM/MM},$$

where $E_{QM}$ includes the electronic energy of the QM atoms for a given nuclear configuration, $E_{MM}$ describes the classical interactions between the MM atoms, and $E_{QM/MM}$ represents the interaction between the QM and MM atoms. Here, QM energies were calculated using the Self-Consistent-Charge Density Functional Tight-Binding (SCC-DFTB) method [29], as implemented in the CHARMM package [16]. Within its accuracy range of 2–3 kcal/mol, SCC-DFTB has been shown to reproduce the B3LYP/6-31G(d,p) geometries of small hydrocarbons [30,31] and to give the correct ordering of relative energies of conformations of small peptides [32,33]. The QM/MM computations of XAO reported here were performed with a QM region consisting of the peptide (149 atoms containing 396 electrons). MM atoms were treated using the charmm36 all-atom topology and parameter files [15] and the TIP3P model for water molecules [21].

### 2.4. Calculating Theoretical Scattering Profiles and Effective R${}_{\mathrm{gyr}}$ Values

The ideal, geometry-based R${}_{\mathrm{gyr}}$ value of a molecule differs from the effective measured value in solution since, under experimental conditions, the solvated molecule is surrounded by a layer of water. For this reason, the theoretical scattering profile is calculated for several computed molecular geometries in order to simulate experimental conditions in which the solvated molecule is surrounded by water. Here, we used 100 randomly chosen simulated structures from the production dynamics. From the scattering profile, the effective R${}_{\mathrm{gyr}}$ can be calculated and compared with experimental values. For this, the CRYSOL software (version 28)was used with all the input parameters set to their default values [34]. The theoretical scattering profile (ln[Intensity] vs. scattering${}^{2}$) is then used in a Guinier analysis to obtain the R${}_{\mathrm{gyr}}$.

## 3. Results and Discussion

### 3.1. Dihedral Angle Distribution

**Figure 2:**
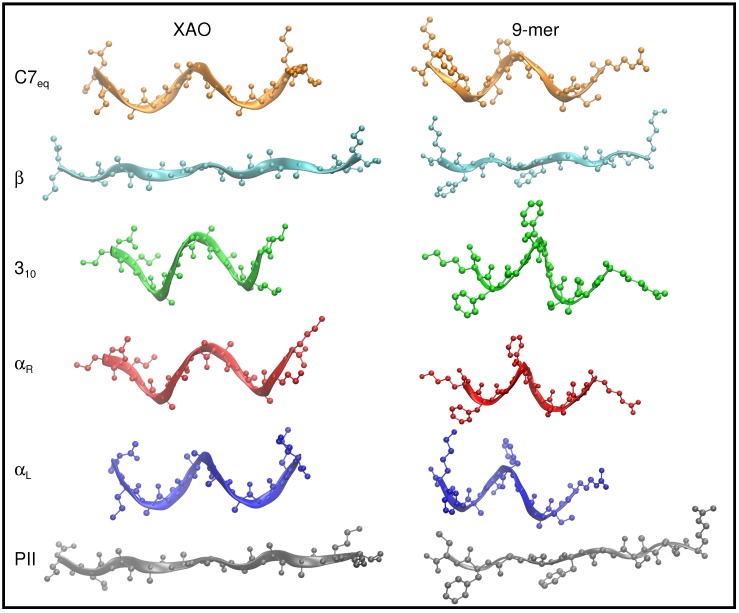
The six peptide conformers, C7${}_{\mathrm{eq}}$ ($\Phi =-120^{\circ }$, $\Psi =50^{\circ }$) (orange), $\alpha_{L}$ ($\Phi =65^{\circ }$, $\Psi =40^{\circ }$) (blue), $\alpha_{R}$ ($\Phi =-65^{\circ }$, $\Psi =-40^{\circ }$) (red), 3${}_{10}$ ($\Phi =-60^{\circ }$, $\Psi =-30^{\circ }$) (green), β ($\Phi =-120^{\circ }$, $\Psi =130^{\circ }$) (cyan), and P${}_{\mathrm{II}}$ ($\Phi =-60^{\circ }$, $\Psi =140^{\circ }$) (gray), that span the Ramachandan space, are depicted schematically for XAO (**left**) and the 9-mer (**right**). For these illustrations, all Φ-Ψ dihedral angles of the peptide backbones are set to the ideal geometry for each of these six conformers. The peptide backbones are drawn with a ribbon representation to highlight geometric differences. Hydrogen atoms are omitted for clarity.

The Φ-Ψ angle space sampled by XAO and the 9-mer was divided into six basins that span the Ramachandran plot (see Table 1 and Figure 2 for representative conformers of C7${}_{\mathrm{eq}}$ (orange), $\alpha_{L}$ (blue), $\alpha_{R}$ (red), 3${}_{10}$ (green), β (cyan), and P${}_{\mathrm{II}}$ (gray)).

**Figure 3:**
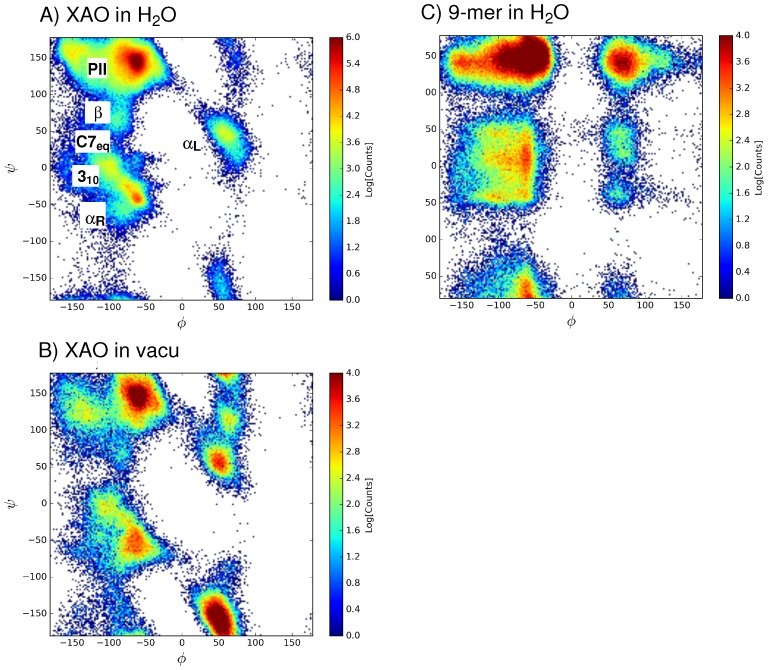
The distribution of Φ-Ψ angles sampled by (**A**) XAO in water (production data collected from 900 ns MD); (**B**) XAO in vacuo (production data collected from 200 ns MD); and (**C**) the 9-mer in water (production data collected from 200 ns data) are shown.

The distribution of Φ-Ψ angles sampled by XAO and the 9-mer in water over 900 ns and 200 ns, respectively, was calculated (shown in Figure 3A,C, respectively). For comparison, the Φ-Ψ angles sampled by XAO over 200 ns in vacuum are shown in Figure 3B. For XAO, each of the 11 Φ-Ψ torsional angle pairs of adjacent XAO peptide residues, was evaluated and assigned to a region of the Ramachandran plot based on the basin classification shown in Table 1. An analogous analysis was carried out for the eight Φ-Ψ pairs of the 9-mer. The total population of each basin was tabulated, and relative populations could be assigned (Table 1). The free energy difference between each basin is calculated according to:

(2) $$F=-k_{B}T\ln\left(N_{b}/N_{ref}\right).$$

In Equation (2), *b* refers to the dihedral angle basin, $N_{b}$ is the number of conformations found in one dihedral angle basin, and the reference basin, $N_{ref}$, is the most populated basin; $k_{B}$ is the Boltzmann constant and *T* is the temperature (300 K).

In comparison with the Ramachandran plot of the 9-mer (Figure 3C), the XAO dihedral angle distribution (Figure 3A) shows a narrower distribution of points in the region Φ = −180${}^{\circ }$ to 0${}^{\circ }$ and Ψ = −50${}^{\circ }$ to 50${}^{\circ }$, as well as in the region Φ = 0${}^{\circ }$ to +180${}^{\circ }$ and Ψ = 100${}^{\circ }$ to 150${}^{\circ }$. One explanation for this difference may be the polyalanine nature of the XAO peptide, XXAAAAAAAOO (X = diaminobutyric acid, A = alanine, O = ornithine), which contains seven consecutive alanine residues, whereas the 9-mer (Arg-Pro-Pro-Gly-Phe-Ser-Ala-Phe-Lys) has no repeating amino acid pattern. A recent report on MD simulations of the same duration (i.e., 200 ns) of Ala${}_{5}$ with several force fields, including charmm36, shows a nearly identical Ramachandran plot for the central residues [17]. This alanine chain may enable a pattern of repeating hydrogen bonds, thus stabilizing more regular secondary structure patterns in the Φ = −180${}^{\circ }$ to 0${}^{\circ }$ half of the torsional space. In the 9-mer, the presence of Pro and Gly, highly flexible due to the absence of steric clashes between backbone and side chain atoms, may allow for the broad population of secondary structures in the $0^{\circ }<\Phi <+180^{\circ }$ and Ψ > 100${}^{\circ }$ region. Nonetheless, in the case of the 9-mer, moderate shifts in population are observed in the second half of the MD trajectory (see convergence analyses in Supplementary information), indicating that longer MD simulations are likely required to reach stable population distribution.

A population analysis (Table 1) of the sampled basins reveals that the P${}_{\mathrm{II}}$ basin has the highest relative population (53%) of the six basins, followed by the β basin with 20% of the ensemble population. The least populated dihedral angle basin is C7${}_{\mathrm{eq}}$ with only 4% of the total population. For the 9-mer, an analogous analysis (Table 2) shows that, as with XAO, the P${}_{\mathrm{II}}$ basin has the highest relative population (44%) of the six basins. However, the second most populated basin is the 3${}_{10}$ basin (with 17%), followed by the β basin (15%). The least populated basin for the 9-mer is the left-handed $\alpha_{L}$ geometry with only 4%. To check the effect of aqueous solvent on the population distribution, a similar population analysis was carried out for the distribution of Φ-Ψ angles sampled by XAO in vacuo. From a comparison of Figure 3A,B and Table 1, a noticeable difference is the significantly larger relative population of left-handed helices ($\alpha_{L}$), as well as a decrease in the P${}_{\mathrm{II}}$ population. This shift in conformational sampling in Figure 3B may be attributed to the lack of aqueous solvent molecules that stabilize the more extended P${}_{\mathrm{II}}$ conformation.

To understand why the 3${}_{10}$ geometry is energetically more stable in the 9-mer than in XAO, we examined the populations of all eight Φ-Ψ pairs in the 9-mer individually. The largest 3${}_{10}$ populations exist in $\Phi_{2}$-$\Psi_{2}$ (∼25%), between Pro and Gly, and in $\Phi_{7}$-$\Psi_{7}$(∼25%), between Ala and Phe. The remaining dihedral angle pairs, except for $\Phi_{4}$-$\Psi_{4}$ between Gly and Phe, which exhibits a high degree(∼40%) of $\alpha_{R}$ geometry, exist predominantly in the P${}_{\mathrm{II}}$ geometry. The relatively bulky side chains of Pro and Phe, especially located adjacent to the highly flexible Gly residue, likely are responsible for the sampling of Φ-Ψ space that is energetically less stable for XAO.

From the distribution of the peptide ensemble amongst the six basins, relative free energy differences can be calculated according to Equation (2) and are listed in Table 1 and Table 2. The energy differences, relative to the most populated basin P${}_{\mathrm{II}}$, range from ∼0.6 kcal to 1.4 kcal/mol at 300 K. It is worthwhile noting that the relative populations of conformational basins, i.e., secondary structure assignment, depends on the partitioning of the Φ-Ψ grid. In a recent paper by Mansiaux et al., the authors investigate the extent of P${}_{\mathrm{II}}$ secondary structure based on a range of secondary structure assignment methods [35]. Applying the widely used DSSP program [36], the authors use the following rules to define a residue as having a P${}_{\mathrm{II}}$ geometry: (1) the residue is recognized by the DSSP program as having a “coil” structure based solely on hydrogen bonding patterns; (2) the residue belongs to at least two consecutive residues labeled as “coil”; (3) the dihedral angles Φ-Ψ are within $\pm 29^{\circ }$ of the canonical definition of $\Phi =-75^{\circ }$ and $\Psi =+145^{\circ }$ [35]. In this work, populations are based on the following dihedral angle assigment for P${}_{\mathrm{II}}$: −180${}^{\circ }$ < Φ < 0${}^{\circ }$ and 135${}^{\circ }$≤Ψ≤ 180${}^{\circ }$. We tested the Φ-Ψ criteria of Mansiaux et al. by changing the lower bound on the Ψ range from 135${}^{\circ }$ to 115${}^{\circ }$, effectively decreasing the area of the neighboring β basin. This Ψ cut-off increases the relative P${}_{\mathrm{II}}$ population from 0.534 to 0.677 while decreasing the β population from 0.202 to 0.068. Here, each of the 11 XAO residues has been analyzed discretely, without regard for the conformation of neighboring residues.

**Figure 4:**
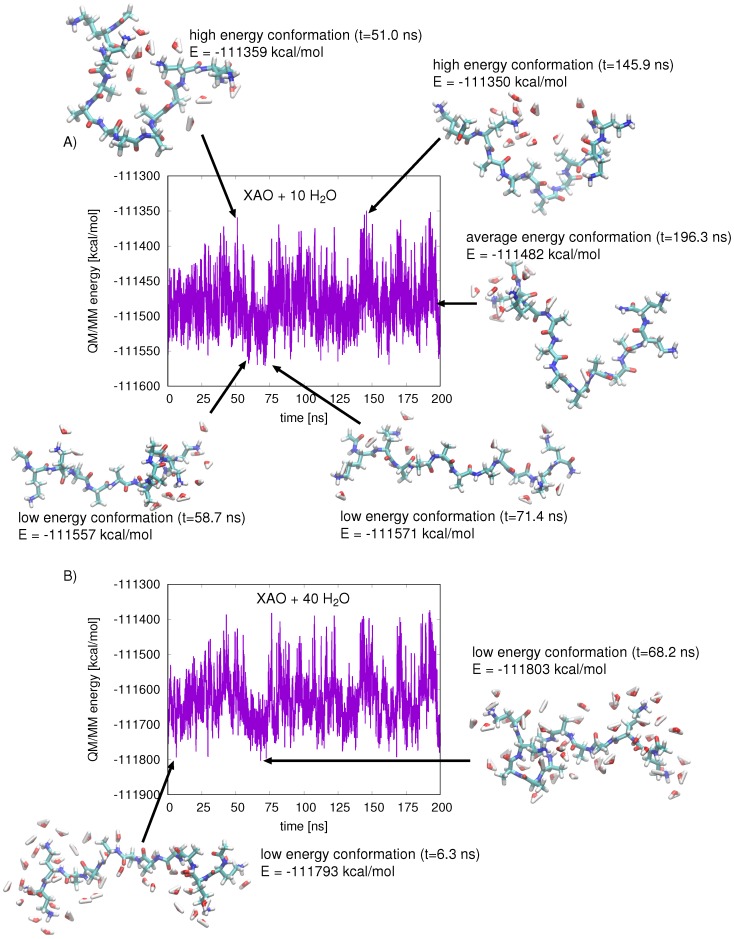
(**A**) the QM/MM energy of XAO and ten closest water molecules is plotted for snapshots taken from production data collected over 200 ns of MD. The geometries of XAO and 10 closest water molecules corresponding to high energy, average energy, and low energy states are shown; the lowest-energy conformation (t = 71.4 ns) shows maximum peptide extension; (**B**) the QM/MM energy of XAO and 40 closest water molecules is plotted for snapshots from production data collected over 200 ns of MD; two representative low-energy geometries are shown.

Relative free energies obtained from the population analysis were compared with average QM/MM energies calculated for each basin of the Ramachandran plot. Snapshots were taken from the 900 ns classical MD simulation of XAO at regular intervals (snapshot/0.05 ns). The total QM/MM energy of the extracted system, containing the peptide and ten closest water molecules, was calculated next, in which the XAO peptide comprises the QM region (Figure 4A). The energies for each conformation were tabulated and average values could be assigned to each Φ-Ψ basin of the Ramachandran plot. These energies, although not QM/MM energy minima, reflect the enthalpic stability of the peptide–solvent conformation. By correlating corresponding geometries to the calculated QM/MM energies, one can rationalize what interactions are responsible for stabilizing the peptide–solvent interactions. A comparison of the XAO geometries (shown schematically in Figure 4A) from snapshots of low energy regions and high energy regions illustrates the interactions with solvent molecules that affect the peptide stability; a structure representative of the average QM/MM energy is shown for comparison. In general, extended XAO geometries that maximize exposure to solvent molecules are energetically stabilizing, whereas compact structures are destabilizing. Indeed, a free energy change of +0.65 kcal/mol (at 298 K) per residue is associated with alanine helix formation due to entropy loss [4]. Additionally, the separation of the charged end groups X (diaminobutyric acid) and O (ornithine) affects the electrostatic energy of the protein and the arrangement of solvent molecules. In the high energy conformations, the compact arrangement of the peptide pushes the charged end groups together, resulting in unfavorable repulsive interactions. The low energy conformations, on the other hand, separate the charged end groups and stabilize the energy. Increasing the number of solvent molecules around the peptide stabilizes its energy, especially for more compact geometries, as the solvent shields the peptide from the destabilizing electrostatic interactions of its positively charged side chains (Figure 4B).

### 3.2. End-to-End Distance, R${}_{\mathrm{gyr}}$

**Figure 5:**
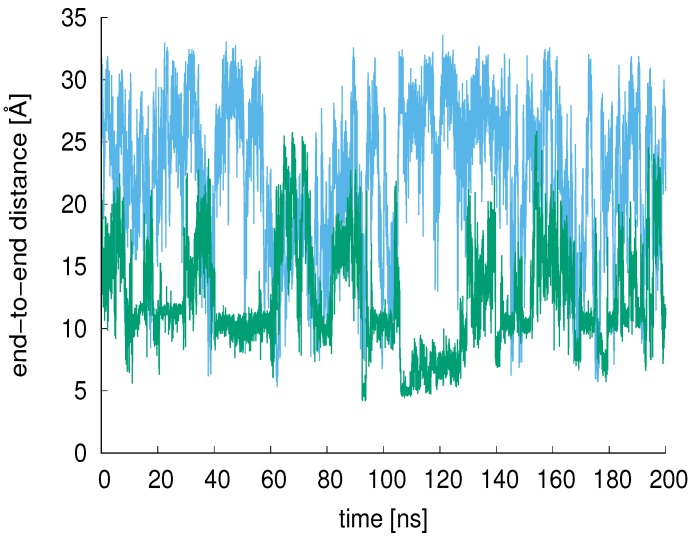
The end-to-end distances of the XAO peptide (blue) and 9-mer (green) are shown for 200 ns MD in aqueous solution. The average XAO end-to-end distance is 22.6 Å (calculated from 900 ns simulation period); the average 9-mer end-to-end distance is 12.4 Å.

The end-to-end distance of the XAO peptide and 9-mer were calculated over the simulation time and compared (200 ns comparison shown in Figure 5). The average XAO end-to-end distance is 22.6 Å (calculated from 900 ns simulation period); the average 9-mer end-to-end distance is 12.4 Å. For a pure P${}_{\mathrm{II}}$ structure, one would expect an end-to-end distance of 32 Å [10].

**Figure 6:**
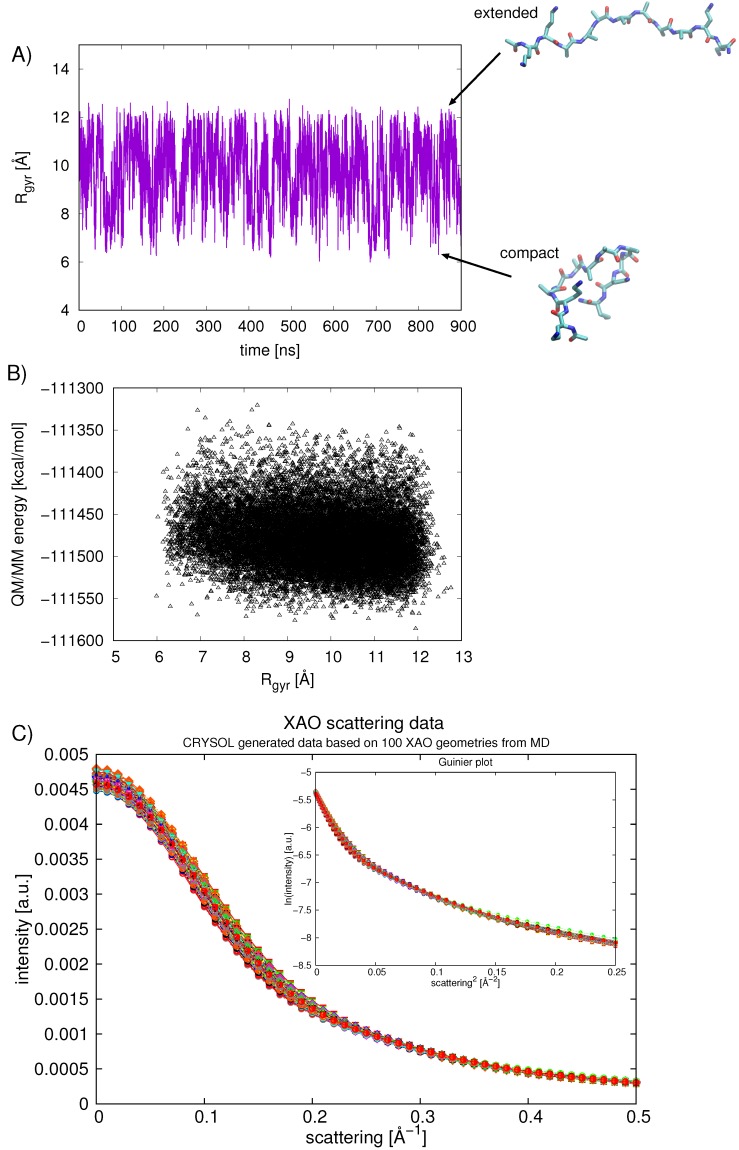
(**A**) the R${}_{\mathrm{gyr}}$ [Å] of XAO calculated over the 900 ns of classical MD; (**B**) the calculated QM/MM energy [kcal/mol] of the XAO peptide is correlated with the R${}_{\mathrm{gyr}}$ [Å]; (**C**) intensity (arbitrary units) versus scattering (Å${}^{-1}$) is plotted for 100 randomly chosen structures from MD simulations of XAO. Inset shows the Guinier plot of ln(intensity) vs. scattering${}^{2}$.

The compactness of the XAO peptide in aqueous solution was further analyzed over the 900 ns of simulation by calculating the R${}_{\mathrm{gyr}}$ (Figure 6). The average calculated R${}_{\mathrm{gyr}}$ value is 9.7 ± 1.4 Å(average over first 450 ns is 9.75 Å and average over second 450 ns is 9.70 Å). The smallest R${}_{\mathrm{gyr}}$ sampled is around 6.5 Å and the largest is approximately 12.2 Å (shown for two representative XAO conformations in Figure 6A). This value approaches the R${}_{\mathrm{gyr}}=11.6$ Å for an all-trans P${}_{\mathrm{II}}$ conformation and 13.0 Å for a fully extended XAO conformation [6]. In other words, XAO visits regions of extended geometries but on average is somewhat more compact. A plot showing the calculated R${}_{\mathrm{gyr}}$ versus the QM/MM energy of the XAO peptide (Figure 6B) indicates a correlation between larger R${}_{\mathrm{gyr}}$ values and lower energies.

To compare our calculated R${}_{\mathrm{gyr}}$ values with both experimental values as well as computed values reported previously in the literature, we calculated the scattering profiles for 100 randomly chosen XAO structures (Figure 6C). The Guinier fit [fit of a straight line in ln(int) vs. s${}^{2}$ ] refers to the analysis of small angle X-ray scattering (SAXS) data that is valid at very small scattering angles, s → 0. In this range, the Guinier analysis allows for the determination of the R${}_{\mathrm{gyr}}$ according to:

(3) $$I\left(s\right)=I\left(0\right)\exp(-\frac{1}{3}{\left(R_{\mathrm{gyr}}\times 2\times \pi \times s\right)}^{2}).$$

In practice, the Guinier analysis tends to be a decent approximation for the regime that 2*π*s*R${}_{\mathrm{gyr}}$< 1 [37]. A fit of the calculated data results in R${}_{\mathrm{gyr}}=7.8\pm 0.2$ Å, in reasonable agreement with the value obtained from SAXS experiments, 7.4±0.5 Å ([7]) and from six ns MD simulations 7.06±0.96 Å ([6]). However, Zagrovic et al. caution against the interpretation of data from conformational averaging [7]. NMR measurements, vibrational spectroscopy, and Raman spectroscopy deliver time- and ensemble-averaged data [7]; these experimental data may misinterpret or in fact over-estimate the amount of P${}_{\mathrm{II}}$ structure present. Based on our combined analysis of the R${}_{\mathrm{gyr}}$ from the MD and scattering data, we suggest that XAO indeed explores a range of conformations and is not confined to the P${}_{\mathrm{II}}$ basin. Nonetheless, the data indicate that the peptide is energetically most stable when its geometry is extended, predominantly in a P${}_{\mathrm{II}}$ conformation.

## 4. Conclusions

The aim of our present investigation was to analyze the propensity of small peptides to exist in a P${}_{\mathrm{II}}$ conformation in aqueous solution. Here, we studied the behavior of two model systems, the 11-residue XAO and a 9-mer. Previous computational and experimental studies have presented mixed evidence regarding the extent of P${}_{\mathrm{II}}$ within an ensemble of XAO peptides. Our analyses of Ramachandran plot populations, end-to-end distances, and R${}_{\mathrm{gyr}}$ values support a model in which XAO is stabilized in an extended conformation. This geometry is found in both the P${}_{\mathrm{II}}$ and β basins of the Ramachandran plot. Average conformational energies, calculated using a QM/MM energy function, indicate that the potential energy differences between basins are at most on the order of ∼5 kcal/mol. Free energy differences of less than 2 kcal/mol, obtained through a simple population analysis of each of the six basins, also support the notion of dynamical interconversion between peptide conformers that are enthalpically and entropically stabilized in extended conformations.

It is worth noting that secondary structure predictors, including DSSP [36], have historically under-assigned the P${}_{\mathrm{II}}$ secondary structure, favoring instead α-helices, β-sheets, and turns; the prevalence of the P${}_{\mathrm{II}}$ conformation among protein and peptide secondary structures may in fact be more widespread than previously thought [35]. In our study, we used the charmm36 force field to study the conformations sampled by two small peptides. As Φ-Ψ sampling can vary depending on the force field, the results presented here should be considered as a representative study of peptide transitions within the framework of the charmm36 force field. Current efforts in designing polarizable force fields will undoubtedly lead to more sophisticated molecular energy surfaces that will better describe conformational transitions in peptides and proteins.

## Figures and Tables

**Table 1:** The relative populations of XAO geometries from simulations in water are listed for six Φ-Ψ geometry basins in Ramachandran plot; populations obtained from simulations in vacuo are listed below in parentheses. Free energy differences [kcal/mol], obtained from a population analysis $F=-k_{B}T\ln\left(N_{b}/N_{ref}\right)$, between the six sampled geometries are listed. The last column lists average QM/MM energies [kcal/mol] calculated from snapshots of XAO and the ten nearest water molecules, extracted from the 900 ns MD simulation. The Φ-Ψ dihedral angles (given in degrees) correspond to the C${}_{i-1}$–N${}_{i}$–C${}_{\alpha i}$–C${}_{i}$ and N${}_{i}$–C${}_{\alpha i}$–C${}_{i}$–N${}_{i+1}$ atoms, respectively, of adjacent peptide residues.

Geometry	Φ	Ψ	Relative Population (In Vacuo)	Free Energy Difference [kcal/mol]	Average QM/MM Energy [kcal/mol]
ine					
P${}_{\mathrm{II}}$	−180 < Φ < 0	135 ≤ Ψ ≤ 180	0.534	0.00	−111,479
			(0.420)		
ine					
β	−180 < Φ < 0	50 ≤ Ψ < 135	0.202	0.58	−111,479
			(0.147)		
ine					
$\alpha_{R}$	−180 < Φ < 0	−180 ≤Ψ < −25	0.126	0.86	−111,475
			(0.150)		
ine					
3${}_{10}$	−180 < Φ < 0	−25 ≤Ψ < 0	0.062	1.28	−111,475
			(0.035)		
ine					
$\alpha_{L}$	0≤ Φ < −180	−180 ≤Ψ≤ 180	0.041	1.53	−111,478
			(0.220)		
ine					
C7${}_{\mathrm{eq}}$	−180 < Φ < 0	0 ≤ Ψ < 50	0.035	1.62	−111,478
			(0.027)		

**Table 2:** The relative populations of 9-mer geometries are listed for six Φ-Ψ geometry basins in Ramachandran plot; free energy differences [kcal/mol], obtained from a population analysis $F=-k_{B}T\ln\left(N_{i}/N_{ref}\right)$, between the six sampled geometries are listed. The last column lists average QM/MM energies [kcal/mol] calculated from snapshots of the 9-mer and the ten nearest water molecules, extracted from the 200 ns MD simulation. The Φ-Ψ dihedral angles (given in degrees) correspond to the C${}_{i-1}$–N${}_{i}$–C${}_{\alpha i}$–C${}_{i}$ and N${}_{i}$–C${}_{\alpha i}$–C${}_{i}$–N${}_{i+1}$ atoms, respectively, of adjacent peptide residues.

Geometry	Φ	Ψ	Relative Population	Free Energy Difference [kcal/mol]	Average QM/MM Energy [kcal/mol]
ine					
P${}_{\mathrm{II}}$	−180 < Φ < 0	135 ≤ Ψ ≤ 180	0.440	0	−111,092
ine					
3${}_{10}$	−180 < Φ < 0	−25 ≤Ψ < 0	0.167	0.58	−111,101
ine					
β	−180 < Φ < 0	50 ≤ Ψ < 135	0.147	0.65	−111,092
ine					
$\alpha_{R}$	−180 < Φ < 0	−180 ≤Ψ < −25	0.105	0.86	−111,084
ine					
C7${}_{\mathrm{eq}}$	−180 < Φ < 0	0 ≤ Ψ < 50	0.099	0.89	−111,096
ine					
$\alpha_{L}$	0≤ Φ < −180	−180 ≤Ψ≤ 180	0.042	1.41	−111,078

## Supplementary Materials

The supplementary materials are available online.

## Abbreviations

The following abbreviations are used in this manuscript:

QM/MM	quantum mechanics/molecular mechanics
MD	molecular dynamics
XAO	X${}_{2}$A${}_{7}$O${}_{2}$, X = diaminobutyric acid, A = alanine, and O = ornithine
