# Supplementary material for: Exploring Peptide–Solvent Interactions: A Computational Study
Source: Molecules. 2018 Sep 14;23(9):2355. doi: 10.3390/molecules23092355 (PMC6225229; doi:10.3390/molecules23092355)
Supplement: Supplementary file 1 [file molecules-23-02355-s001.pdf]

1 Supplementary information: Convergence tests

To check convergence of the MD production data for XAO and the 9-mer, the total MD trajectories were divided into two halves, for XAO from $0 < \text{t} < 450$ ns and from $450 < \text{t} < 900$ ns, and for the 9-mer from $0 < \text{t} < 100$ ns and from $100 < \text{t} < 200$ ns. The properties that have been analyzed, *i.e.* Φ,Ψ angle distribution, R_{gyr}, and end-to-end distances, have again been re-analyzed for each half of the trajectory and are compared below.

1.1 XAO

The Φ,Ψ distributions are analyzed and shown in Figure 1 and relative populations are listed in Table 1. R_{gyr} average values (Figure 2) between first half, 9.75 Å, and second half, 9.70 Å, show a difference of 0.05 Å, approximately 0.5 % deviation. End-to-end average values (Figure 3) between first half, 22.7 Å, and second half, 22.5 Å, show a difference of 0.2 Å, approximately 0.9 % deviation.

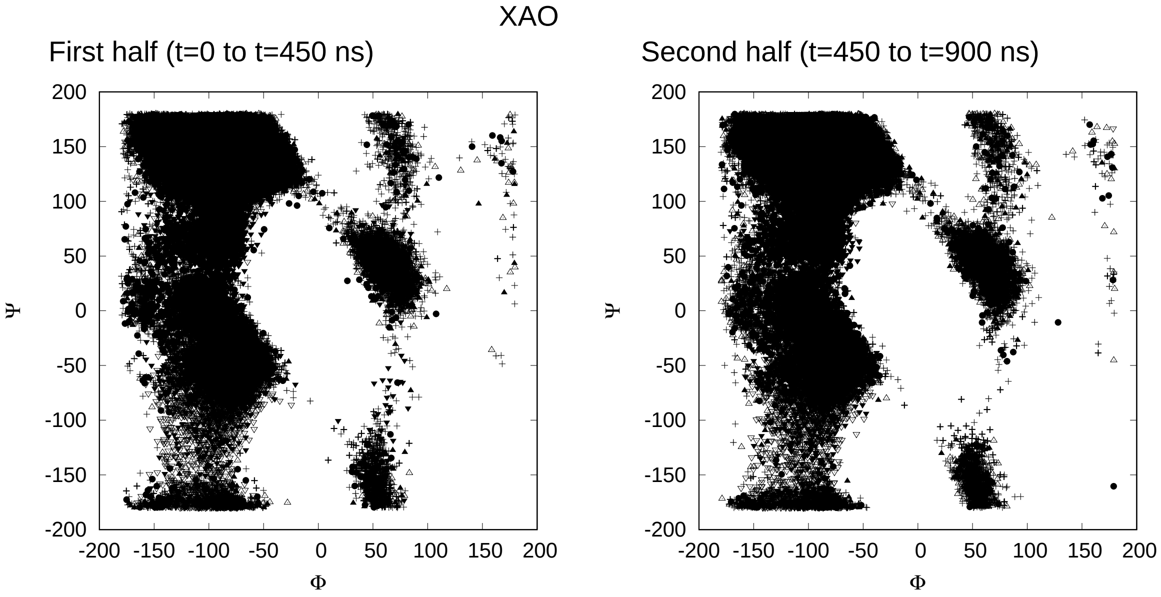

Figure 1: Comparison of Ramachandran plots for first half (0→450 ns) and second half (450→900 ns) of MD simulation of XAO.

geometry	Φ	Ψ	Relative population		
			Total time $0 < \mathrm{t} < 900$ ns	First half $0 < \mathrm{t} < 450$ ns	Second half $450 < \mathrm{t} < 900$ ns
P_{II}	$-180 < \Phi < 0$	$135 \leq \Psi \leq 180$	0.534	0.535	0.532
β	$-180 < \Phi < 0$	$50 \leq \Psi < 135$	0.202	0.201	0.203
α_R	$-180 < \Phi < 0$	$-180 \leq \Psi < -25$	0.126	0.129	0.122
3_{10}	$-180 < \Phi < 0$	$-25 \leq \Psi < 0$	0.062	0.060	0.065
α_L	$0 \leq \Phi < -180$	$-180 \leq \Psi \leq 180$	0.041	0.040	0.042
$C7_{eq}$	$-180 < \Phi < 0$	$0 \leq \Psi < 50$	0.035	0.035	0.035

Table 1: The relative populations of XAO geometries are listed for six Φ-Ψ geometry basins in Ramachandran plot. Average values obtained from the first and second trajectory halves, respectively, of the 900 ns MD simulation are listed for comparison. The Φ-Ψ dihedral angles (given in degrees) correspond to the C_{i-1}–N_i–$C_{\alpha i}$–C_i and N_i–$C_{\alpha i}$–C_i–N_{i+1} atoms, respectively, of adjacent peptide residues.

1.2 9-mer

The Φ,Ψ distributions are analyzed and shown in Figure 4 and relative populations are listed in Table 2. R_{gyr} average values (Figure 5) between first half, 7.04 Å, and second half, 6.80 Å, show a difference of 0.05 Å, approximately 3.5 % deviation. End-to-end average values (Figure 6) between first half, 13.4 Å, and second half, 11.4 Å, show a difference of 2.0 Å, approximately 17.5 % deviation.

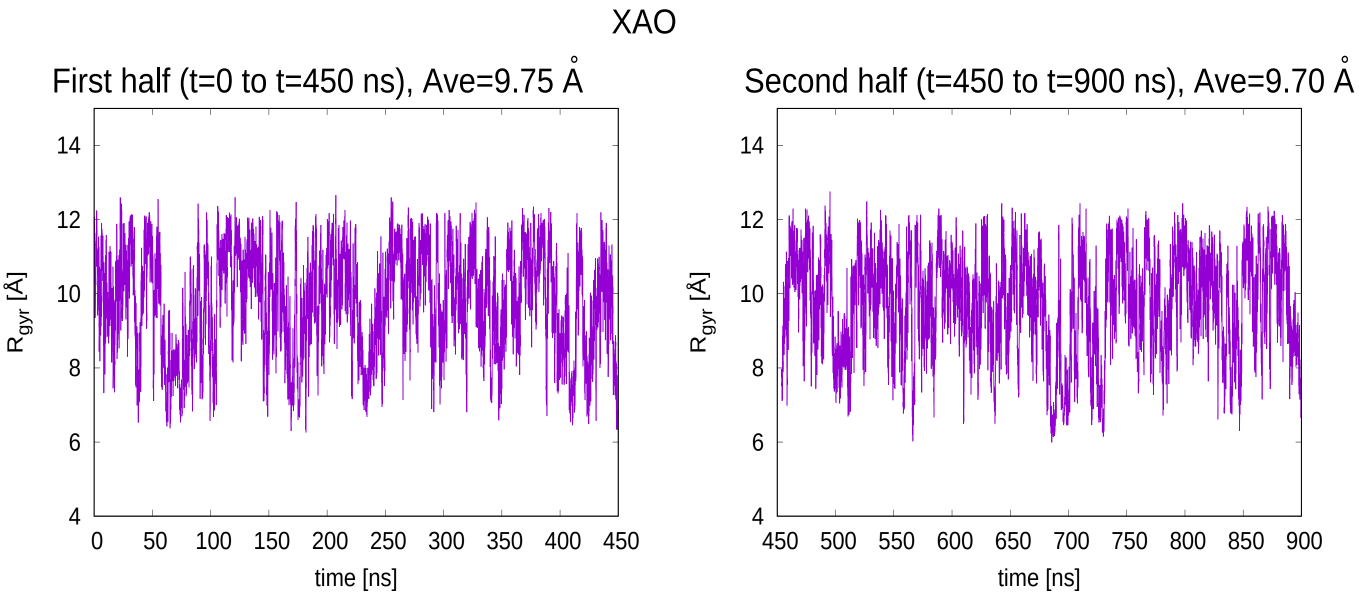

Figure 2: Comparison of R_{gyr} (Å) for first half (0→450 ns) and second half (450→900 ns) of MD simulation of XAO.

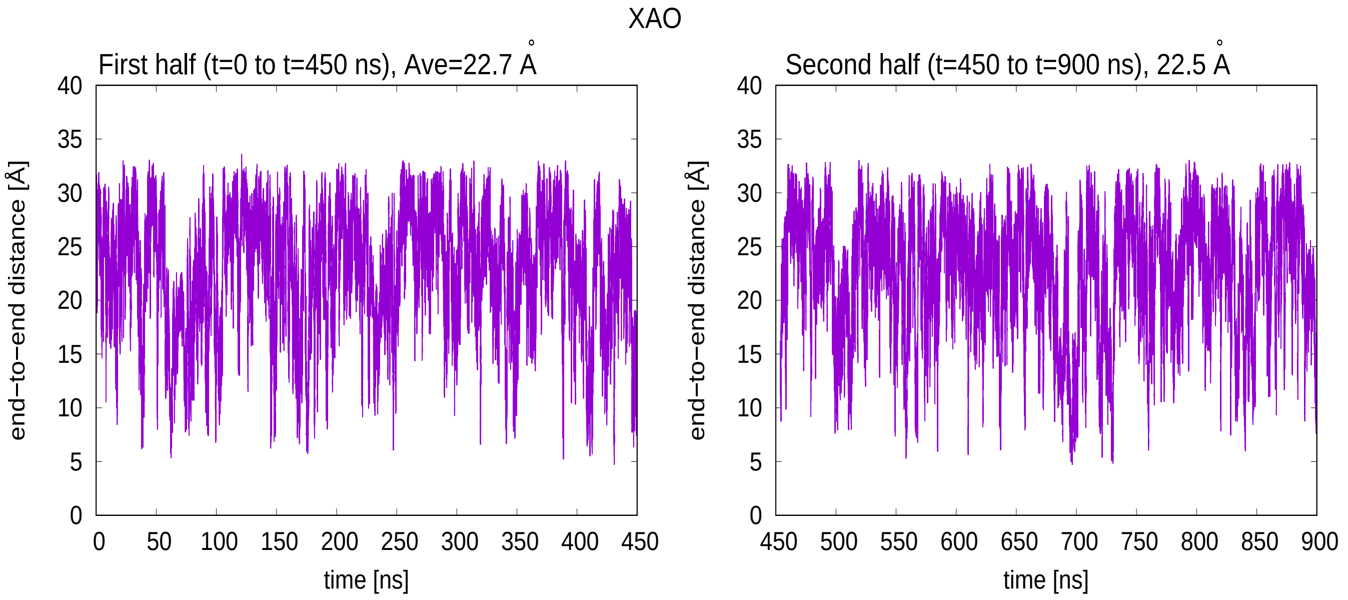

Figure 3: Comparison of end-to-end distances (Å) for first half (0→450 ns) and second half (450→900 ns) of MD simulation of XAO.

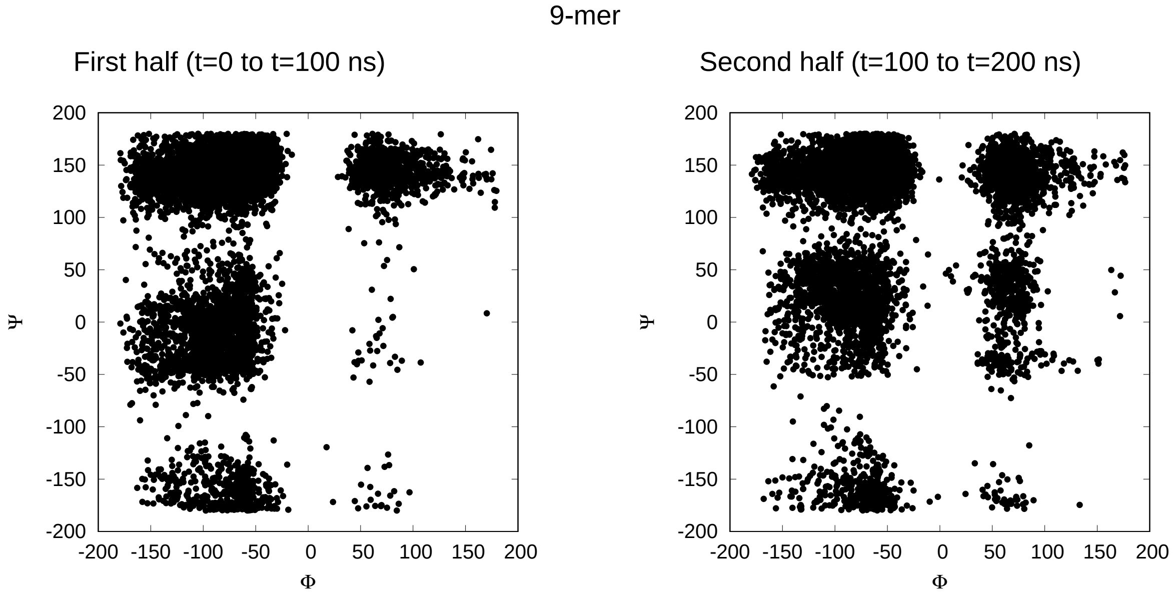

Figure 4: Comparison of Ramachandran plots for first half (0→100 ns) and second half (100→200 ns) of MD simulation of 9-mer.

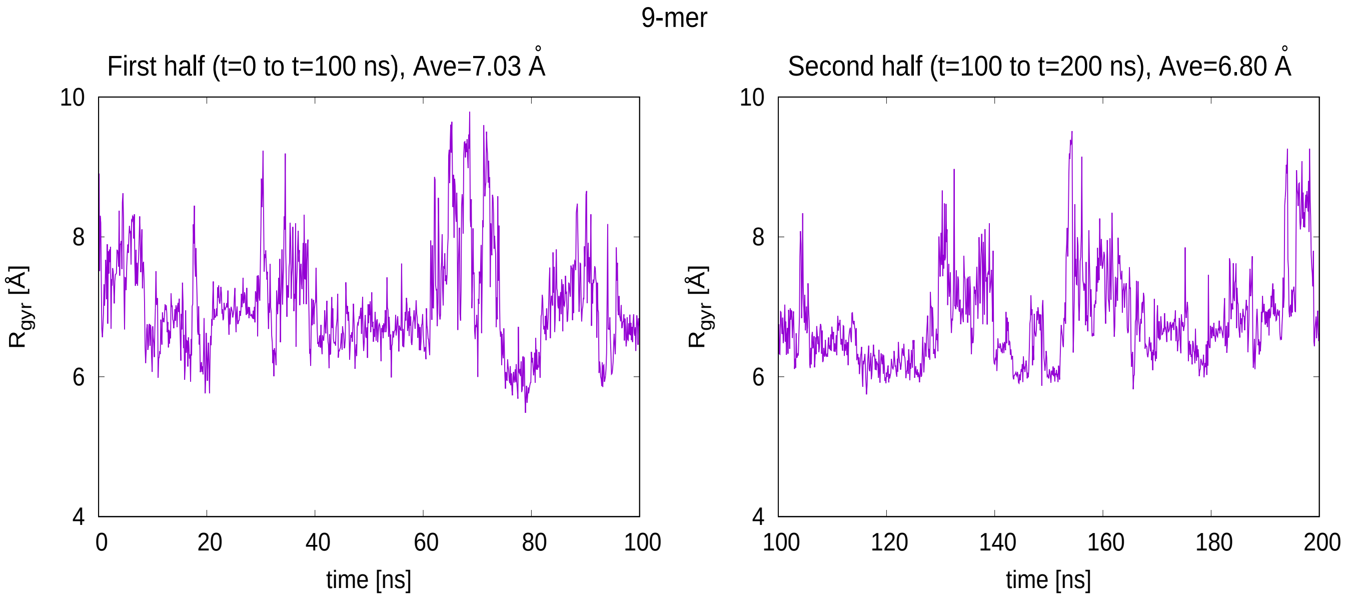

Figure 5: Comparison of R_{gyr} (Å) for first half (0→100 ns) and second half (100→200 ns) of MD simulation of 9-mer.

**geometry**	Φ	Ψ	**Relative population**		
			Total time $0 < \mathrm{t} < 200$ ns	First half $0 < \mathrm{t} < 100$ ns	Second half $100 < \mathrm{t} < 200$ ns
P_{II}	$-180 < \Phi < 0$	$135 \leq \Psi \leq 180$	0.440	0.461	0.420
3_{10}	$-180 < \Phi < 0$	$-25 \leq \Psi < 0$	0.167	0.110	0.223
β	$-180 < \Phi < 0$	$50 \leq \Psi < 135$	0.147	0.142	0.153
α_R	$-180 < \Phi < 0$	$-180 \leq \Psi$ ¡ -25	0.105	0.141	0.068
$C7_{eq}$	$-180 < \Phi < 0$	$0 \leq \Psi < 50$	0.099	0.086	0.113
α_L	$0 \leq \Phi < -180$	$-180 \leq \Psi \leq 180$	0.042	0.060	0.023

Table 2: The relative populations of 9-mer geometries are listed for six Φ-Ψ geometry basins in Ramachandran plot. Average values obtained from the first and second trajectory halves, respectively, of the 200 ns MD simulation are listed for comparison. The Φ-Ψ dihedral angles (given in degrees) correspond to the C_{i-1}–N_i–$C_{\alpha i}$–C_i and N_i–$C_{\alpha i}$–C_i–N_{i+1} atoms, respectively, of adjacent peptide residues.

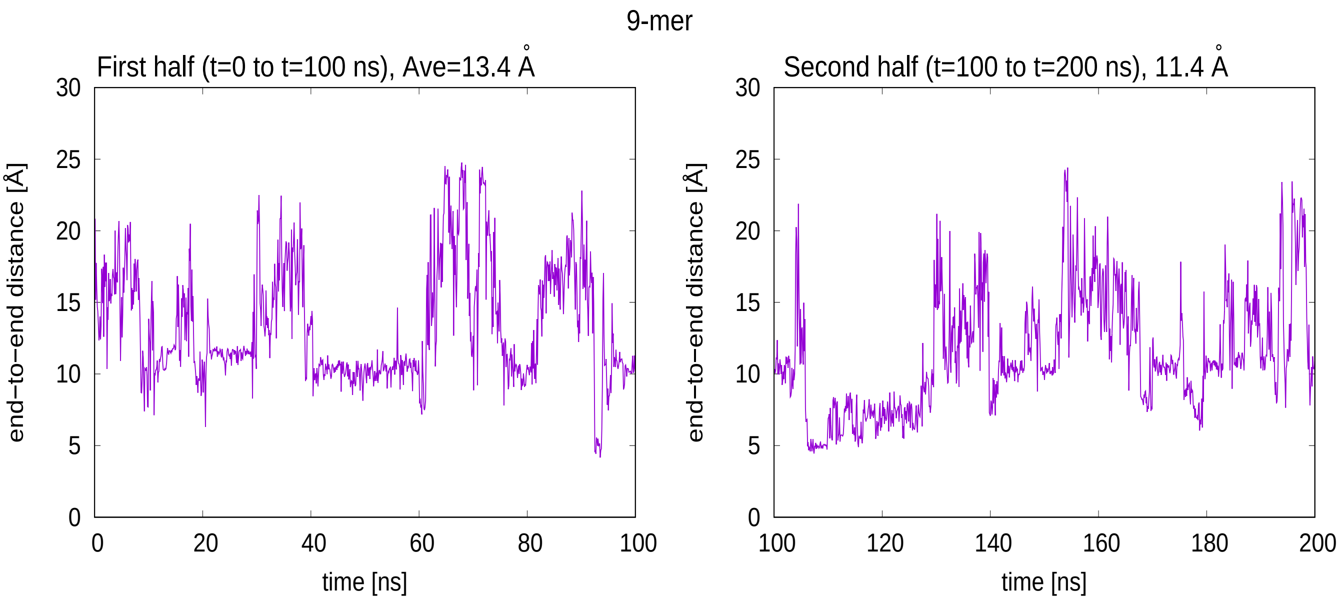

Figure 6: Comparison of end-to-end distances (Å) for first half (0→100 ns) and second half (100→200 ns) of MD simulation of 9-mer.
